# Enhanced Processing of Regrind as Recycling Material in Single-Screw Extruders

**DOI:** 10.3390/polym13101540

**Published:** 2021-05-11

**Authors:** Philipp Thieleke, Christian Bonten

**Affiliations:** Institut für Kunststofftechnik, University of Stuttgart, 70569 Stuttgart, Germany; philipp.thieleke@ikt.uni-stuttgart.de

**Keywords:** extrusion, solids conveying, grooved feed zone, regrind, numerical simulation, discrete element method, superquadrics, elastic-plastic contact model

## Abstract

Regrind processing poses challenges for single-screw extruders due to the irregularly shaped particles. For grooved feed zones, the output is lessened by the reduction of bulk density in comparison to virgin material. Simultaneously, the melt temperature increases, reducing the extruder’s process window. Through experimental investigations on a test stand, a novel feed zone geometry (nominal diameter 35 mm) is developed. It aligns the regrind’s specific throughput with that of virgin material. The regrind processing window is essentially increased. As the solids conveying in the novel feed zone cannot be simulated with existing methods, numerical simulations using the discrete element method are performed. Since plastic deformation occurs in the novel feed zone geometry, a new hysteresis contact model is developed. In addition to spheres, the regrind and virgin particles are modeled as superquadrics to better approximate the irregular shape. The new contact model’s simulation results show excellent agreement with experimental compression tests. The throughput of the extruder simulations is considerably underestimated when using spheres to represent the real particles than when using irregularly shaped superquadrics. Corresponding advantages can be seen especially for virgin material.

## 1. Introduction

**Figure 1:**
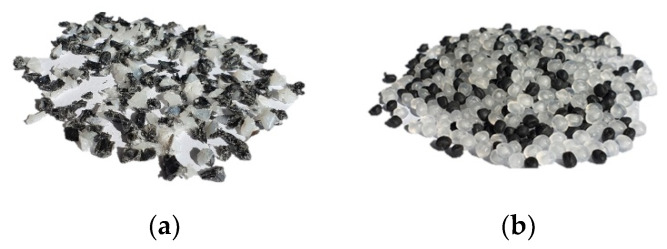
(**a**) Regrind material; (**b**) virgin material granules.

The recycling of plastics will grow considerably in the future. Thereby, the proportion of regrind material processed by single-screw extruders would equally increase. However, the irregular shape of the regrind particles in comparison to virgin material (see Figure 1) becomes a challenge for single-screw extruders.

**Figure 2:**
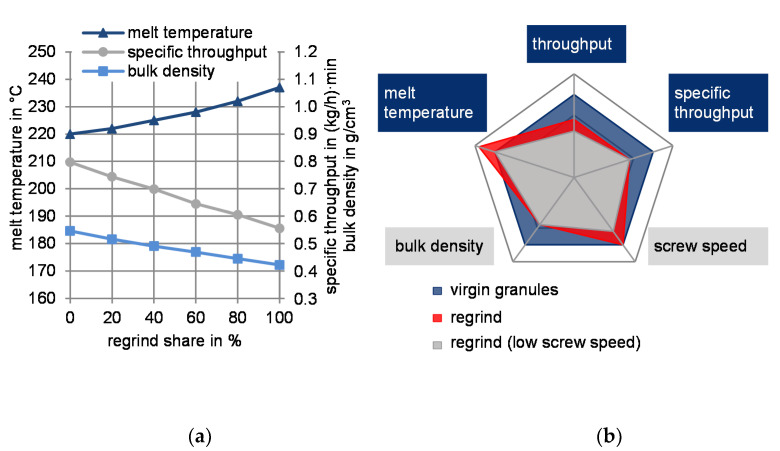
(**a**) Influence of an increased regrind share adapted with permission from [2]. 2021 Michels; (**b**) reduced process window through regrind processing.

When using grooved feed zone extruders, regrind material comprises a lower bulk density, resulting in a reduced specific throughput (throughput per screw revolution). This in turn decreases the extruder’s efficiency [1]. The extruder’s lower specific throughput leads to a higher melt temperature, which devalues the melt quality (Figure 2b, red area). The melt temperatures can be lowered by a reduced screw speed. (Figure 2b, gray area). This shows that regrind processing essentially reduces the extruder’s process window.

An improved feeding process of single-screw extruders has long been the goal of this research field. Hegele [3] examined eccentric filling zones where the granules could not be pushed back into the hopper section. This enhanced the solids conveying of the feed zone, which was also confirmed by further investigations by Potente et al. [4]. 

Recent work regarding asymmetric feed pockets was conducted by Michels [2] and Sikora [5,6]. However, eccentric filling zones are counterproductive for regrinding and reduce the specific throughput [2].

An optimized feed zone geometry for regrind processing can be found in so-called feed pockets. In the filling zone, the barrel’s inner diameter is larger than the nominal diameter. This provides the regrind material more space to flow into the extruder’s filling zone. However, it is recommended that the feed pocket’s design be kept small to prevent stagnation of material [2]. The influence of the angle from the feed pocket diameter to the nominal diameter was investigated by Krämer [7]. The flatter the angle, the higher the specific throughput.

**Figure 3:**
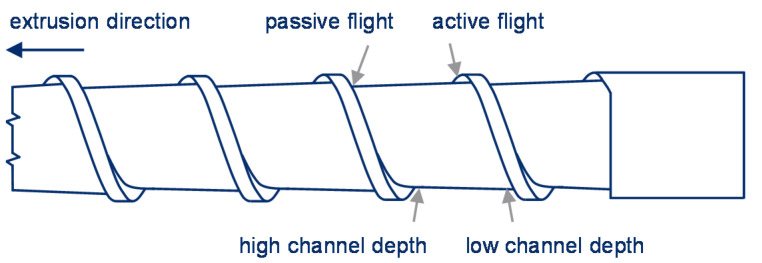
Conical screw channel for improved conveying adapted with permission from [8]. 2021 Rahal.

Based on theoretical considerations, experimental investigations, and calculations carried out, Rahal [8] determined that a maximum effective flight depth must not be exceeded on the active screw flight, as otherwise the conveying efficiency would be negatively influenced. To maintain a sufficient screw channel volume, the screw channel is conical in an axial direction (see Figure 3). 

Other research focused on adjustable means to influence the specific throughput of a single-screw extruder depending on the requirements. Behounek [9] developed a coaxial screw in which the feeding screw’s screw speed was independent of the plasticizing screw. In addition, special feed supports were developed, which are arranged separately from the plasticizing screw and can thus regulate the specific throughput [10]. Furthermore, attempts were made to influence the specific throughput with a variable groove geometry. Corresponding patents can be found in Kautz [11] and Peiffer [12]. However, the developments seen remained in prototype status and were not brought to market maturity [13].

There has been numerous research into calculating the solids conveying throughputs of single-screw extruders. It started with Peiffer’s [14] model of one-dimensional descriptions which was later refined by Kaczmarek [13]. Further essential one-dimensional models were developed by Grünschloß [15] and Schöppner [16]. Hennes [17] established the basis for the two-dimensional description of the throughput that was further developed into a three-dimensional one by Imhoff [18].

**Figure 4:**
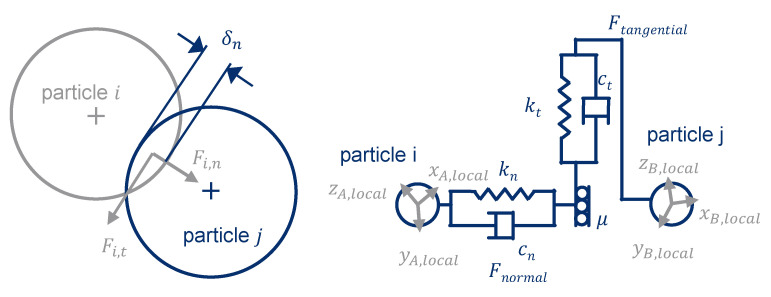
Contact forces at particle collision adapted with permission from [24]. 2021 Trippe.

However, for an efficient three-dimensional consideration of solids conveying processes, the discrete element method (DEM) is used, in which the plastic granules are modeled as spheres. Cundall and Strack [19] developed the DEM in 1979 [20]. Newtonian laws of motion form the basis for the DEM, with particles having three translational and three rotational degrees of freedom. However, the particles are rigid and non-deformable. Therefore, simulation of processes with plastic deformation is challenging. Normal and tangential forces result from particle-particle collisions (see Figure 4). The selected contact law determines the normal and tangential forces, which comprise the spring stiffness k, the damping component c, and the friction µ (see Equations (1) and (2)) [21]. The new position of the particles can be calculated for a corresponding time step size [22]. Basis of the calculation is the virtual overlapping of the colliding particles [23].

(1) $$F_{n}=-k_{n}\;\delta_{n}+c_{n}\;\Delta v_{n\;}$$

(2) $$F_{t\;}=min\;\left\{\left|k_{t\;}\int \Delta v_{t}\;dt+c_{t\;}\Delta v_{t\;}\right|,\mu \cdot F_{n\;}\right\}$$

Moysey and Thomson first used the discrete element method for three-dimensional simulations of a single-screw extruder’s feed zone [25]. Recirculation effects in the filling zone were detected, which had previously been determined in other investigations [26]. Further DEM applications were used to refine the boundary conditions and to optimize the feed zone geometry [27,28,29,30,31]. Other research proved that feed pockets are beneficial for the filling of the screw channel, which is relevant for regrind processing.

**Figure 5:**
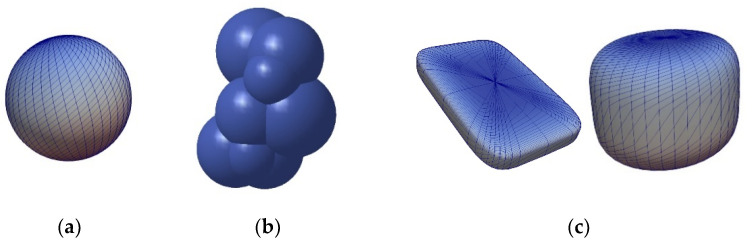
Representation of particle shapes in simulation environment (**a**) sphere; (**b**) multisphere; (**c**) superquadric.

The particles of DEM simulation are generally represented as spheres (Figure 5a), although the real plastic granules are not ideally spherical. Other methods, such as multispheres (Figure 5b), allow for a better approximation of the real particle shape. One particle is made up of several other particles. Amberger et al. used this method in [32] for the approximation of non-convex and arbitrary objects. The disadvantage is a higher simulation time [22]. Finally, the particles can be approximated by superquadrics (Figure 5c). The shape is defined according to Formula (3) [20]. The shape of superquadrics results from a distortion of spheres and ellipsoids. The semi-axis lengths in *x*-, *y*-, and *z*-direction are defined by the parameters *a_Sq_*, *b_Sq_*, and *c_Sq_*. The shape parameters *n_Sq,_*_1_ and *n_Sq_*_,2_ determine how angular the superquadric particle is shaped. Since there is only one particle, simulation time is reduced as compared to multispheres [20].

(3) $$f\left(x\right)\equiv {\left({\left|\frac{x}{a_{Sq}}\right|}^{n_{Sq,2}}+{\left|\frac{y}{b_{Sq}}\right|}^{n_{Sq,2}}\right)}^{\frac{n_{Sq,1}}{n_{Sq,2}}}+{\left|\frac{z}{c_{Sq}}\right|}^{n_{Sq,1}}-1=0\;x={\left(x,y,z\right)}^{T}$$

**Figure 6:**
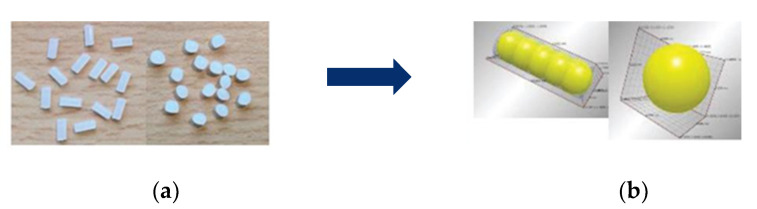
Transfer of the (**a**) real granule shape into (**b**) 3D simulation adapted with permission from [24]. 2021 Trippe.

Leßmann and Schöppner [33] used the multisphere method to approximate the real shape of particles, which were cylinders and lenses (Figure 6). It was found that bulk density simulations comprising the approximation had an essential influence on the result. Leßmann used the examinations in [33] for further simulations of the feed zone with a smooth barrel in [34]. However, the output prediction did not improve. Further research on DEM simulations regarding solids conveying of single-screw extruders and particle shapes can be extracted from [35,36,37,38,39,40,41].

The spring and damper constants can be calculated according to Kloss [42]. The widely used fully elastic contact model is based on Hertz’s [43] work for the description of normal forces through a contact. Mindlin and Deresiewicz [44] extended Hertz’s model for the tangential force calculation. Additionally, the Hertz and Mindlin model is a non-linear elastic model as the spring and damper values are dependent on the particle overlap [42].

**Figure 7:**
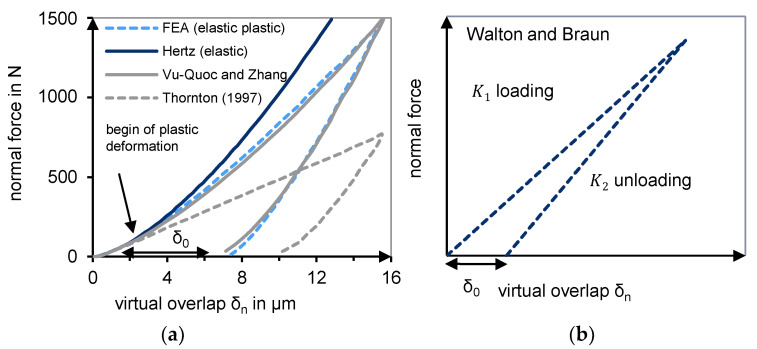
(**a**) Remaining plastic deformation after load adapted with permission from [46]. 2021 Vu-Quoc; (**b**) Contact model of Walton and Braun adapted with permission from [24]. 2021 Trippe.

Unlike purely elastic models (Hertz), elastic-plastic models took into account the remaining deformation after unloading (Figure 7). The models of Thornton–Ning [44,45], VuQuoc–Zhang [46,47], Brake [48], and Walton and Braun [49,50,51] should be named in this context. Walton and Braun’s (Figure 7b) model consists of two different stiffnesses for the loading and unloading phases. The reduced stiffness for the relief phase leads to residual plastic deformation.

The objective of this work is to develop a feed zone geometry that achieves a complete alignment of the specific throughput when processing regrind. Thereby, the process window of the extrusion unit is significantly increased. The geometry should act passively and not influence the specific throughput through adjustable elements. This ensures an easy production-related realization of the feed zone geometry. Since such expected processes can no longer be described with analytical models, the DEM is used to perform solids conveying simulations.

**Figure 8:**
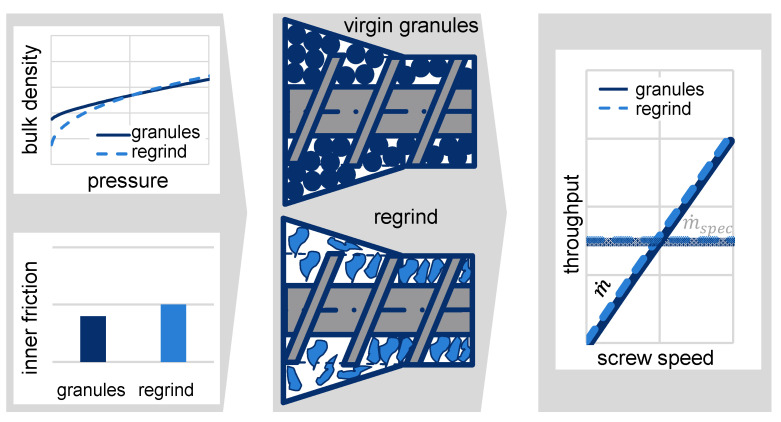
Visualization of the theorem.

**Theorem** **1.** *In terms of compressibility and internal friction, the different bulk material properties of virgin granules and regrinds can be exploited by a compression zone in the single-screw extruder’s feed zone, achieving a full alignment of the specific throughput of virgin granules and regrinds without adjustable elements. This increases the process window for regrind processing in terms of throughput and melt temperature (see Figure 8).*

The following experimental investigations, contact model developments, and results are part of the German Ph.D. thesis of Thieleke [52]. 

## 2. Methods and Experiments

### Experimental Setup

**Figure 9:**
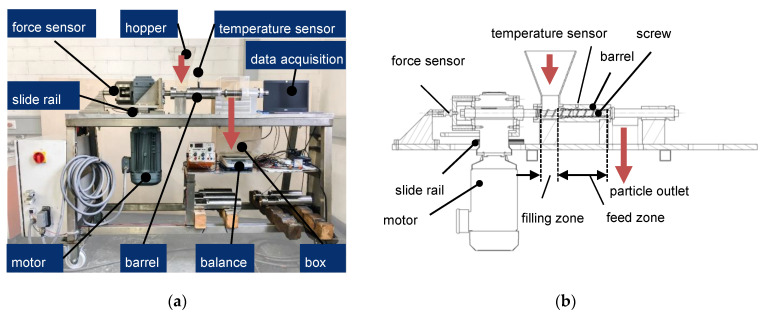
(**a**) Experimental setup; (**b**) sectional view of the test stand.

The experimental investigations aim to examine different feed zone geometries cost-effectively. This is intended to determine the optimal feed zone geometry. For this purpose, a test stand is developed (similar to [53,54]) that only covers the barrel’s feed zone (Figure 9). The test stand was developed within the research project (see funding below, grant number ZF4041120CM7) together with the company Helix GmbH (Winnenden, Germany). The plastic is not molten within the test stand. With the mentioned test stand, it is possible to record the axial force of the screw in the direction of the gearbox. 

**Figure 10:**
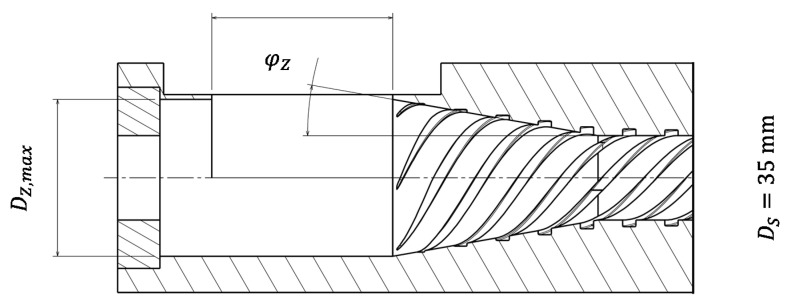
Variation of geometry parameters of the feed zone.

The feed zone geometry of the single-screw extruder was developed based on the assumption that the compressibility and the internal friction of virgin granules and regrind are different. These different bulk properties will be used to align the specific throughput. Therefore, the bulk material must be compressed in the feed zone. The required compression ratio and the exact design of the compression zone are examined using different feed zone geometries. The following parameters of the feed zone geometry are varied (Figure 10).

- Diameter of filling zone D_Z,max_;
- Angle of compression zone φ_Z_;
- Groove design of compression zone.

**Figure 11:**
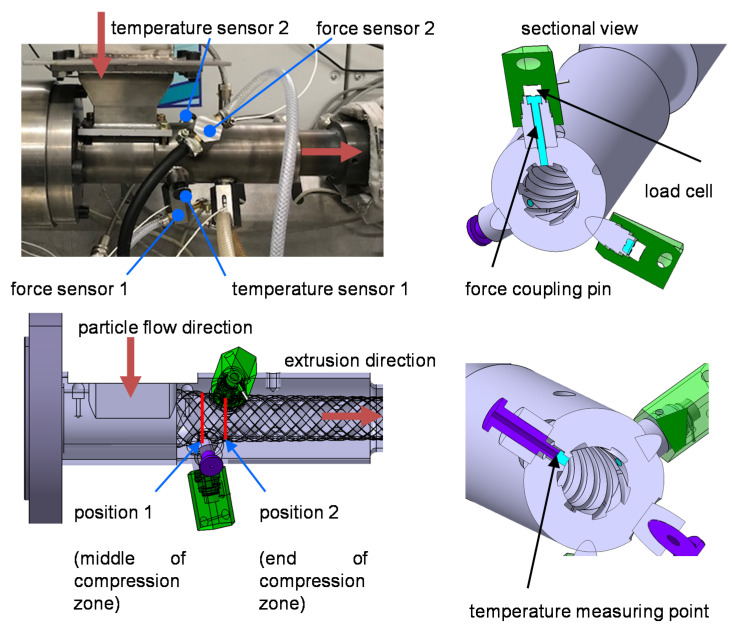
Radial force and temperature measurements at the feed zone.

To evaluate the pressure level within the feed zone, two force sensors are applied in the compression zone (Figure 11). The forces and temperatures are originally recorded in a plasticizing barrel (see Thieleke [52]). The forces are recorded at two axial positions. Sensor 1 is positioned in the middle of the compression zone and sensor 2 at the end. The temperature is recorded at the same axial positions (Figure 11). 

## 3. Materials

For the investigations, polyolefins from the material group high-density polyethylene (PE-HD) and polypropylene (PP) were chosen (Table 1). The selected PE-HD is Lupolen 4261AG UV60005, which is a product of the company Lyonell Basell, Rotterdam, Netherlands. The homopolymer DuPure G 72 TF from the company Ducor Petrochemicals, Rozenburg, Netherlands is used as PP. PE-HD is relevant for the blow molding of hollow parts. Blow molding involves an internal process regrind and is often returned to the process in a middle layer with a regrind share of 100%. Consideration of the PE-HD regrind is also essential for increasing recycling rates for blow-molded post-consumer containers in the future.

PP is increasingly used in flat film production and frequently replaces films that were previously made of polystyrene. Internal process regrind is also produced in flat film production and is returned to the extruder in the form of thin shreds. 

A future range of applications regarding post-consumer articles may include thermoformed food trays. After usage, these thermoformed trays could be ground, washed, and added to the flat film process as regrind. Table 1 also shows the approximated shape of the particles for the implementation in the DEM simulation. To obtain these shapes, the particles were scanned (at least 200) to determine the particle size distribution. An optical analysis defined the shape parameter of the superquadrics *n_SQ_*_,1_ and *n_SQ_*_,2_ (roundness of the particle edges). By scanning the particles, the minimum and maximum ellipsoid diameters were determined using the Fiji software [52]. The weight of all particles and the density were used to calculate the average particle thickness [55]. 

Additionally, the equivalent sphere diameter was determined by measuring the particles’ volume of the superquadrics. The particle size distribution is applied to superquadrics and spheres (see results in Thieleke and Bonten [56]).

**Figure 12:**
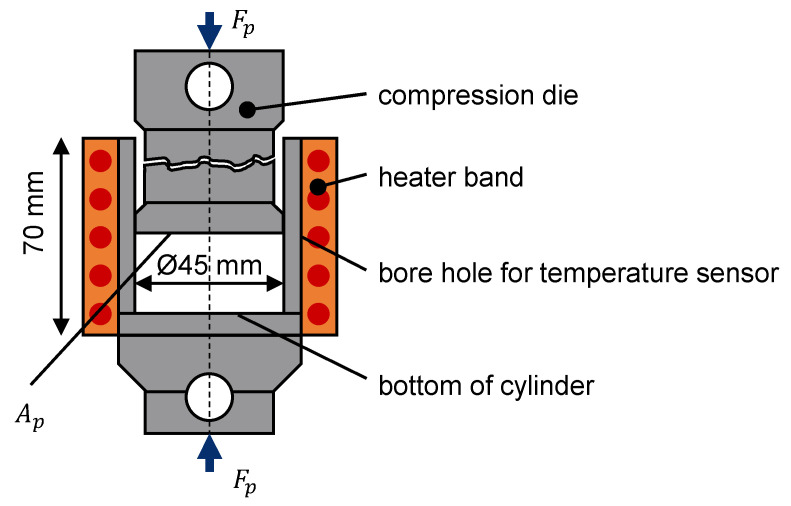
Compression test with a tensile/compression testing machine.

Furthermore, the materials were analyzed for bulk density according to Grünschloß [15] and compression tests (as shown in Figure 12) were conducted to determine its behavior under pressure and temperature (see [57]). An amount of 40 g of each material was inserted into the cup. The selected temperatures (23 °C, 61.5 °C, 100 °C) were set with the heater band. Before being filled, each material was heated up to the corresponding temperature in an oven. After filling, the compression die compacted the material to the chosen pressure levels of 50 bar, 100 bar, and 150 bar.

## 4. Numerical Simulation

The numerical simulation is carried out for the novel feed zone geometry, which aligns the regrind’s specific throughput to that of the virgin material (see Chapter 4. Results). The numerical simulations are performed using version 3.8.0 of the open-source DEM software LIGGGHTS^®^ from DCS Computing GmbH in Linz, Austria. LIGGGHTS^®^ is chosen as the source code since it can be adapted to allow for contact model adjustments. As large plastic deformation occurs in the compression zone, a new contact model is developed for the simulation of the novel feed zone geometry. Existing contact models are not able to realize such large plastic deformations. 

For a better overview, some of the material properties that are ultimately used for the simulation are summarized in Table 2. These parameters are used for virgin and regrind material, as they are material properties not considered for their particle shape. 

For the development of the novel contact model with plastic deformation, the hysteresis contact model of Walton and Braun [50,51] is selected as the basis. This consists of a linear loading phase and a linear unloading phase (cf. Figure 7b). Different slopes of the load and unload lines result in permanent deformation. 

The experimental investigations on the tensile/compression testing machine are used to set up the contact model for the test materials PE-HD virgin, PE-HD regrind, PP virgin, and PP regrind. The deformation behavior is considered dependent on pressure and temperature.

**Figure 13:**
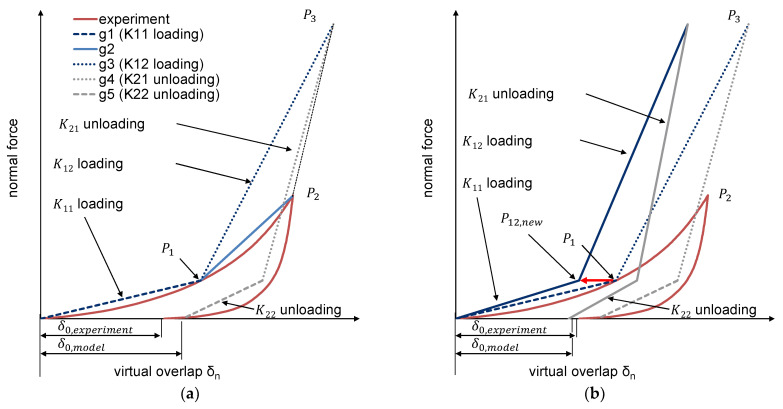
Contact model development. (**a**) starting point (**b**) shift.

The pure linearization of Walton and Braun’s contact model results in a poor fit of the bulk material compression for small forces. In addition, the deformation turns out to be too small for large forces. For this reason, a two-stage hysteresis contact model is selected for the loading and unloading phases (Figure 13a). The compression test of PE-HD virgin material is used to explain the determination. It is carried out equivalently for the remaining three bulk materials. 

Two straight lines (g_1_ and g_2_) define the load curve of the compression test of PE-HD (Figure 13, red curve) up to a maximum force of 20,000 N. The intersection point P_12_ is on the load curve. With numerical approximation, the integral of the two straight lines g_1_ and g_2_ with the load curve is minimized. Another boundary for the numerical approximation is that both integrals of g_1_ and g_2_ with the experimental load curve are equal. This results in P_12_. 

A third straight line (g_3_), which passes through the intersection point P_12_ and the point P_3,_ is defined. P_3_ is described by the density and the volume obtained from the pvT measurement on the respective material at a defined temperature ϑ_2_ measured in the compression zone (see measurement of temperature 2 in Figure 11). The pressure of the pvT curve was 300 bar. Thus, P_3_ denotes an assumed compacted solid bed and represents a maximum bulk density. 

In the loading phase, the two-stage model is formed starting from the straight line g_1_ up to the intersection point P_12_ with the stiffness K_11,_ while the straight line g_3_ begins from the intersection point P_12_ up to the point P_3_ with the stiffness K_12_. This is used to calculate correct forces for small overlaps δ_n_. For large particle overlaps, the second stage prevents the forces from remaining very small. 

For the unloading stage, Walton and Braun’s model is followed. The relation of the coefficient of restitution is used to measure the change in stiffness. This results in the straight line g_4_ with stiffness K_21_. At the same y-value as point P_12_, the second unloading curve starts with another stiffness (g_5_). In comparison to stiffness K_12,_ stiffness K_22_ is also determined via the coefficient of restitution.

The linear equations are established using the experimental compression of the bulk. However, the contact model defines the force/deformation curve for a single particle. For this reason, the intersection point P_12_ is iteratively shifted horizontally (P_12,new_) until the resulting load curve from the numerical simulation of the bulk compression considerably agrees with the experiment’s reference curve (Figure 13b).

The ratio of the stiffnesses K_11_ and K_12_ is kept constant during the iteration. Due to the simulation time, the iterations are performed solely for modeling the particles as spheres. The obtained contact models for the particular bulk material are used for superquadrics as well. The iteration starting values and the selected stiffnesses are listed in Appendix B (Table A1). 

### Validation of the Numerical Simulation

**Figure 14:**
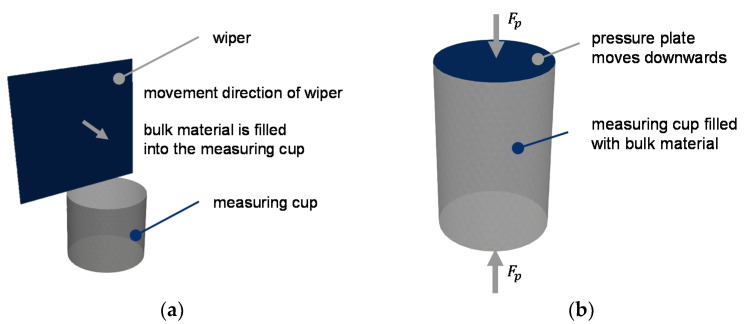
Simulation setup for the (**a**) bulk density; (**b**) compression behavior.

The simulative determination of the bulk density will show if the particle shapes lead to a realistic match. The experimental bulk density measurements serve as a reference. The bulk density simulations are performed in the same way as the DIN EN ISO 60 [58] test. The measuring cup with a depth of 51 mm is filled with granules until it is full. A wiper strips off the protruding particles (Figure 14a). 

After the stripping process, the mass of the particles in the measuring cup is determined. The mass divided by the volume results in the bulk density. The simulations are performed with spheres and superquadrics for virgin material and regrind, respectively. After the particle bulk density simulations, the compression test simulations are conducted. The developed contact models are used for each material. The bulk material is compacted to a maximum force of 20,000 N (approximately 138 bar). The pressure is set by the movable pressure plate, which is force-controlled by a servo command (Figure 14b). In contrast to the experiment, the influence of temperature is passively considered by the developed contact model.

**Figure 15:**
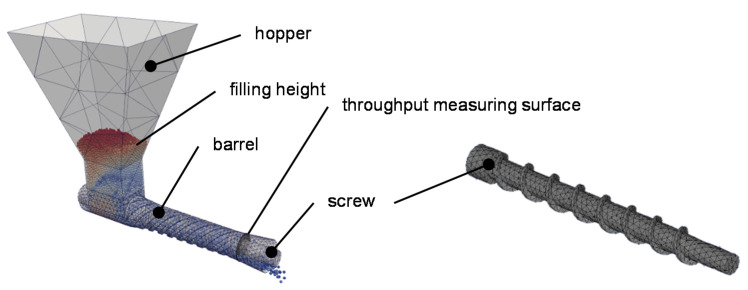
Simulation setup to determine the solids conveying.

The simulation of the single-screw extruder solely considers the solids conveying in the feed zone. It is comparable to the experimental investigation on the test stand. The components have meshed using the meshing software Salome from Open Cascade SAS. The screw and barrel meshing level of detail is set to a maximum element size of 7 mm. The hopper was coarsely meshed. Salome generates a triangle-based surface model in STL format (Figure 15).

In each case, the simulation time is 19.2 s in real-time to obtain a steady state and be able to make reliable statements. In simulative preliminary investigations, it was determined that the selected simulation time is sufficient for a constant throughput.

## 5. Results

The results are divided into two categories: test stand experimental results and DEM simulation results.

### 5.1. Experimental Results of the Feed Zone Geometry Variation

**Figure 16:**
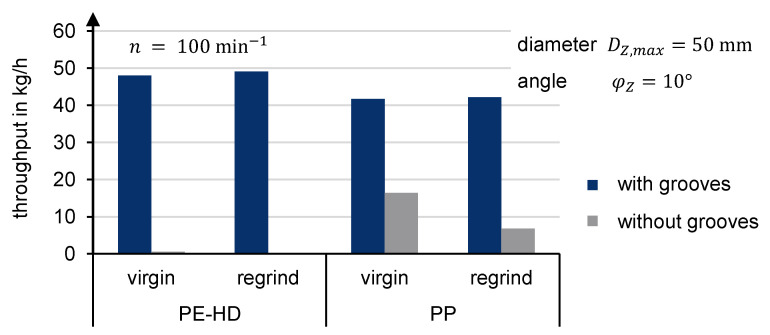
Experimental throughput depending on grooves in the compression zone.

The trials run on the test stand with a grooved and smooth compression zone reveal that the feed zone’s conical area must be grooved to ensure adequate solids conveying (Figure 16). For PE-HD, no conveying takes place when the compression zone is smooth. For PP, an unstable output was observed when the compression zone is without grooves. 

**Figure 17:**
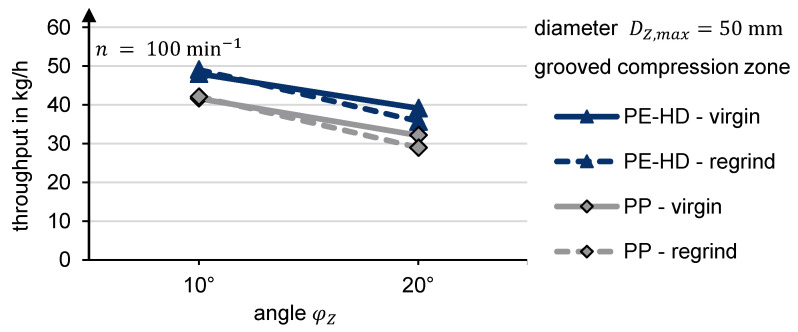
Experimental throughput depending on the angle of the compression zone.

A comparison of the compression zone’s angle variation is shown in Figure 17. The diameter of the filling zone was kept constant at D_Z,max_ = 50 mm. The chart demonstrates that the angle can be used to influence the throughput. A flatter angle increases the throughput. Trials with an angle of 5° achieved an even higher output. However, since the results could not be taken for every material, the data were excluded. These findings already illustrate that a 10° angle can be used to achieve an alignment of the specific throughputs. That alignment of the specific throughputs of regrind and virgin material with a grooved compression zone is also shown in Figure 16 for D_Z,max_ = 50 mm and φ_Z_ = 10°. 

**Figure 18:**
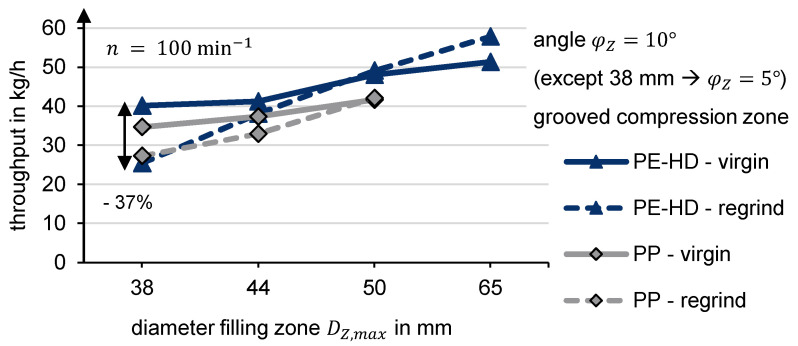
Experimental throughput depending on diameter extension filling zone.

Further comparisons of the filling diameter indicate that a compression zone can influence the difference in throughput between regrind and virgin material. D_Z,max_ = 38 mm represents a standard feed zone with a difference of 37% (Figure 18). A larger filling diameter reduces the throughput difference. At D_Z,max_ = 50 mm, the throughputs are almost equal. In contrast to virgin material (D_Z,max_ = 65 mm), a larger diameter leads to an even higher throughput of regrind.

**Proof** **of** **Theorem 1.** With these findings, the aforementioned theorem is proven. With a new type of compression zone in the feed zone, the different bulk material properties of virgin granules and regrind material, in terms of internal friction and compressibility, are exploited to achieve a complete alignment of the specific throughput within a compression zone. □

For the considered PE-HD and PP bulk materials, the optimum feed zone geometry with a nominal diameter of 35 mm includes a grooved compression zone, a filling diameter of D_Z,max_ = 50 mm, and an angle of φ_Z_ = 10°.

The pressure and temperature measurements in the novel feed zone geometry are decisive for the DEM’s new contact model. The pressure level is evaluated by the axial force measurement and temperature measurements at the test stand. Additionally, the measurement setup in Figure 11 is used to determine the precise force and temperature measurements.

**Figure 19:**
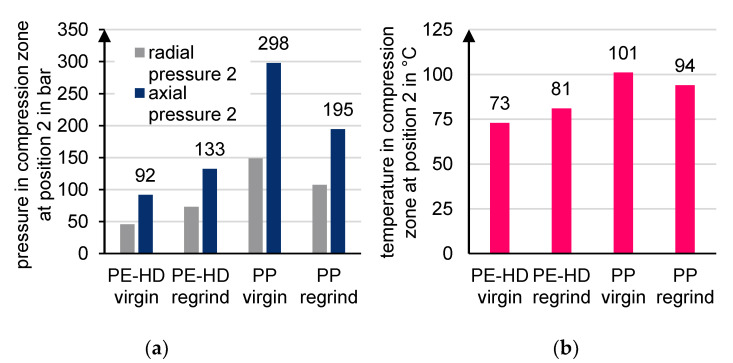
(**a**) Radial and axial forces and (**b**) temperatures in the feed zone.

To obtain the axial pressure from the radial pressure, the pressure anisotropy k must be considered for bulk solids. According to [2,59,60], k_virgin_ = 0.5 is chosen for the moving bulk material consisting of virgin material. Since the pressure anisotropy coefficient k for regrind is larger than for virgin material, k_regrind_ = 0.55 is determined for the regrind [2]. From the measurement of the radial force, the radial pressure at position 2 can be calculated using the cross-section of the force application pin and the respective anisotropy coefficient (Figure 19a). In addition, Figure 19b shows the measured temperature at position 2. The results of the pressure and temperature measurements depict quite similar tendencies.

### 5.2. Result of the Validation of the Numerical Simulation

#### 5.2.1. Bulk Density Simulation

**Figure 20:**
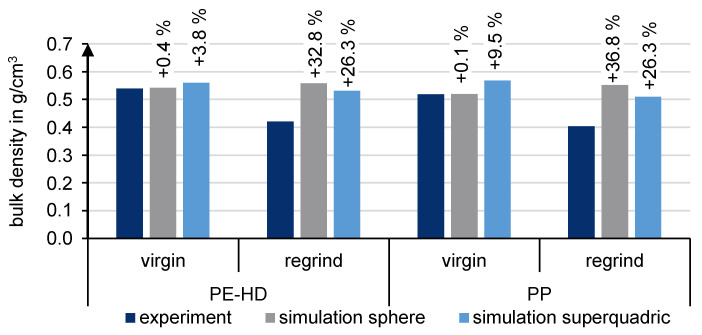
Bulk density comparison between experiment and simulation.

The numerical determination of the bulk density reflects how well the approximation with spheres and superquadrics achieved the real particle shape of the virgin granules and the regrinds. For this purpose, Figure 20 compares the results of the experiment and simulation. It can be seen that spheres are better suited to represent the real bulk density in the case of virgin granules. The deviation is less than 1%. With the superquadrics’ more accurate approximation of the real particle shape, the bulk density for PE-HD and PP is slightly overestimated. 

The simulative determination of the bulk density of regrind remains a challenge. The bulk density of spheres and superquadrics is significantly overestimated. However, superquadrics reduce the discrepancy between experiment and numerical simulation. Superquadrics achieve better results due to the platelet shape. However, all superquadric regrind particles are platelet-shaped in the simulation environment. In reality, these are rather irregularly shaped, making them more likely to interlock and create hollow spaces between particles. In the future, different shapes of regrind particles may be included to achieve enhanced results for superquadrics, especially for regrind. 

In terms of bulk density simulation, spheres can be recommended for virgin granules. For regrind materials, the approximation of the particle shape with superquadrics leads to better results. 

#### 5.2.2. Compression Test Simulation

**Figure 21:**
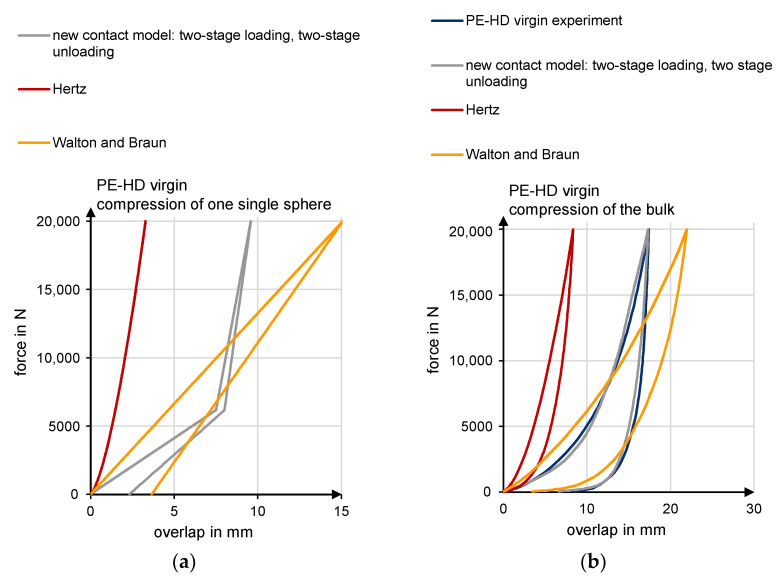
(**a**) General compression behavior of the contact models for one sphere; (**b**) compression behavior of the contact models for the bulk according to a compression test.

The developed contact model is checked for suitability through the compression test simulations. A comparison of different state-of-the-art contact models is made (Figure 21). The newly developed elastic-plastic contact model is compared to those of Hertz and Walton and Braun.

Figure 21a shows the force/deformation curve for the compression of a single sphere with a diameter of 45 mm. These results are used to describe the deformation behavior of a single particle, which defines the respective contact model. In Figure 21b, the force/deformation curve is plotted for the compression of the bulk virgin material of PE-HD. In this case, the particles are defined as spheres. 

If Hertz’s (red) purely elastic contact model is used, the simulated force/deformation curve of the single sphere is identical to the load and unload curve. The particles’ rearrangement effects produce a hysteresis curve. However, there is no remaining deformation, and the load and unload curves are much steeper compared to the other models. 

This results in a poor approximation to the experimentally determined curve (dark blue curve) in Figure 21b. The poor agreement is due to the model’s purely elastic behavior, with no representation of plastic deformation. As a result, the model is extremely stiff. An increasing force results in deformations that are too small compared to reality. Furthermore, the temperature is not considered. The experimental curve (dark blue) of PE-HD represents a temperature of 73 °C.

When using Walton and Braun’s (orange) elastic-plastic contact model, the different stiffnesses for loading and unloading result in a residual deformation. It shows small deformations for small forces and extremely large deformations for larger forces. The approximation to the experiment is also not successful with this model.

The two-stage approach of Walton and Braun’s (gray) hysteresis model, which was developed here, yields an excellent agreement with the obtained experimental curve. Due to the two-stage loading phase, the deformation course of the bulk material can be efficiently reproduced. Furthermore, the temperature influence can be optimally considered.

**Figure A1:**
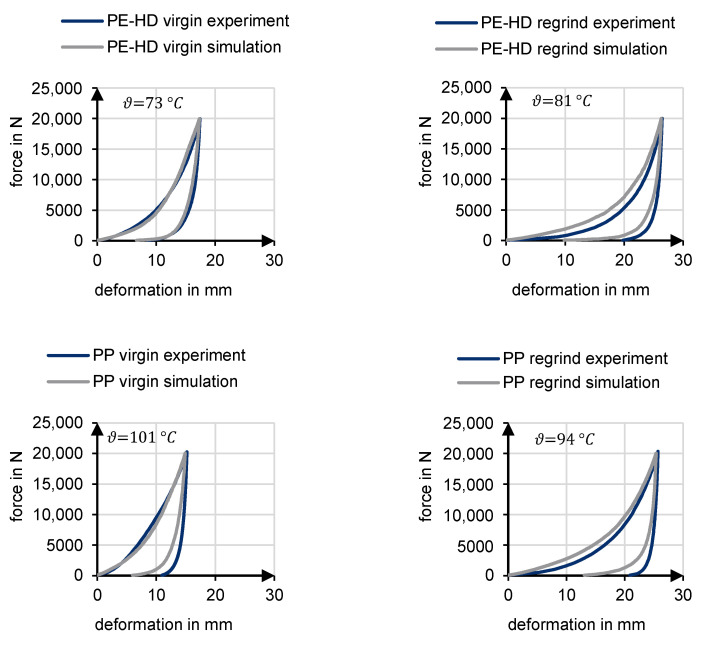
Comparison between experimental and simulation data of the compression test.

The additionally simulated force/deformation curves for PE-HD and PP in comparison to the experimental curves are shown in Appendix A (Figure A1). In comparison to virgin material, a larger deviation can be observed for regrind. The reason for this is that the model cannot be set arbitrarily soft, and the determined curves must be considered optimum for a stable numerical calculation.

#### 5.2.3. Extruder Simulations

**Figure 22:**
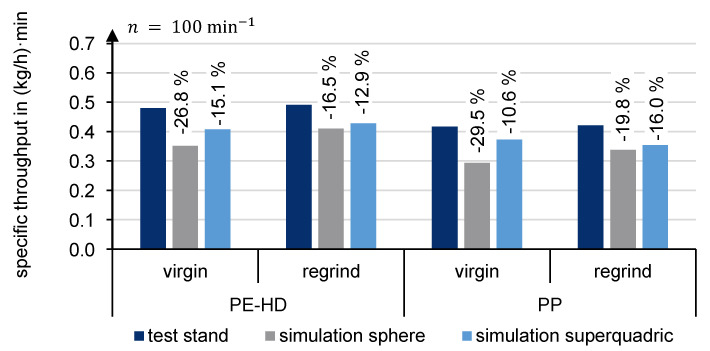
Mass throughput comparison between experiment and simulation.

The results of the extruder simulations are summarized in Figure 22. It shows the specific throughput of PE-HD and PP for virgin and regrind at a screw speed of 100 min^−1^. The simulations were performed with spheres and superquadrics, using the newly developed contact model with two-stage loading and two-stage unloading phases.

It appears that the specific throughput is generally underestimated. When spheres are used, the deviation is significantly larger than with superquadrics. In the case of PE-HD virgin material, for example, although the bulk density is optimally represented by spheres and the compression curve highly corresponds to the compression experiment curve, the numerical extruder simulation reflects a deviation of 26.8%. In the simulation, the particles in the compression zone are assumed to slide away from each other. Therefore, fewer particles are conveyed into the compression zone and ultimately compressed.

The specific throughput for PE-HD virgin material simulated with superquadrics depicts a deviation of 15.1%. The non-spherical shape probably prevents early slippage between the particles in the compression zone.

It is particularly apparent that superquadrics reproduce the alignment of the specific throughput for virgin and regrind material, which was also obtained in the experiment. This result is valid for PE-HD and PP.

In summary, numerical simulation with DEM generally under-predicts the throughputs of the novel feed zone geometry. Since the bulk density and contact model achieve outstanding agreements with the compression simulation, especially for the virgin granules, it can be assumed that either the remaining deformation (after plastic deformation) or the conveying in the compression zone is not yet completely and realistically represented.

Concerning the conveying behavior of the bulk materials, further experimental investigations on flowing bulk materials could help to optimize the throughput in the numerical simulation. Particularly for regrind approximated by superquadrics, the actual bulk density in the moving state could differ significantly from the static measurements, since the platelet-shaped particles would form more hollow spaces between each other. This would compensate for the discrepancy between experimentally and simulatively determined bulk density in the static case. The work of Hennes [17], which investigates the pressure anisotropy on moving bulk solids, could serve as a model for future research on moving bulk solids in terms of bulk density and conveying behavior.

According to Walton and Braun’s new hysteresis model, a remaining deformation is not stored after complete unloading. Consequently, if a new contact arises, the loading starts again from the beginning. This will probably have the greatest influence on the discrepancy between experiment and simulation throughputs. Another approach pursued by DCS Computing GmbH, Linz, Austria, and the team of Christoph Kloss [61,62] is to reduce a particle’s diameter after plastic deformation. Thus, each particle’s remaining deformation is stored. However, this method is currently reserved for the premium version of LIGGGHTS^®^ and is not implemented in the open-source version. It is expected that this approach will reduce the current gap between experimentally and simulatively determined throughputs.

Furthermore, pressure and temperature rise in the compression zone. So far, a constant value is used for the temperature, which is reflected in the contact model. In further investigations, the resulting frictional heat could also be calculated to better implement the temperature in the simulation. In line with this approach, reference can be made to Trippe’s research [24].

## 6. Conclusions and Outlook

The investigations reveal the relevance of regrind processing in single-screw extrusion. A novel feed zone geometry allows for the complete alignment of the specific throughput of regrind and virgin material. It is achieved using an extruder with a nominal screw diameter of 35 mm without adjustable elements. For this purpose, a new type of compression zone is used in the feed zone of the extruder. Through experimental investigations on a specially developed test stand, it was possible to determine the optimum feed zone geometry with regard to the diameter of the filling zone D_Z,max_, the groove design, and the angle φ_Z_ of the compression zone. 

**Figure 23:**
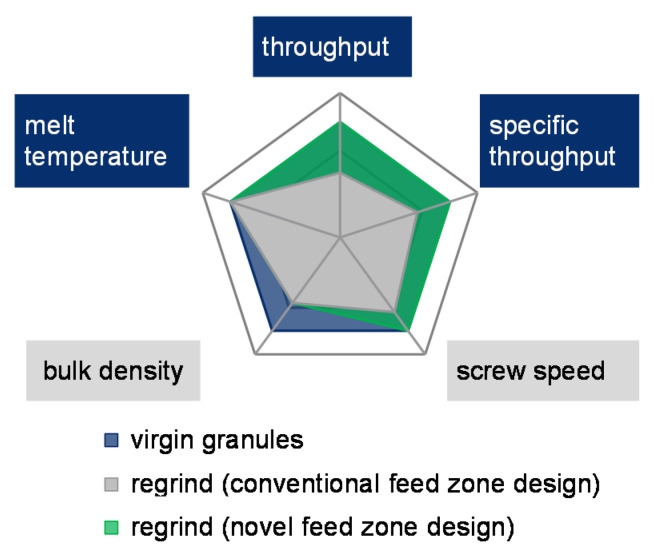
Increased process window for the processing of regrind material.

In contrast to the respective virgin material, a complete alignment of the specific throughput is achieved for both PE-HD and PP regrind. The increased specific throughput simultaneously increases the absolute throughput. It substantially expands the regrind processing process window as summarized in Figure 23. The new process window for regrind is defined by the green area.

Superquadrics, rather than pure spheres, can better represent the real particle shape of regrind in numerical simulations through DEM. This shows advantages in reference to bulk density measurements and extruder throughput measurements. Based on compression tests on real bulk materials, a new hysteresis contact model is set up for the DEM following the approach of Walton and Braun. A two-stage loading and two-stage unloading process are included in the model. Compression tests with temperature influence were used to calibrate the model, which accurately represents plastic deformation. When using the new contact model for the extruder simulations, the throughputs are generally underpredicted. 

To improve the predicted throughput by numerical simulation using DEM, the particles’ remaining deformation through plastic deformation could be performed using a particle radius reduction. This approach is already implemented by DCS Computing GmbH, Linz, Austria, and the team of Christoph Kloss in the premium version of LIGGGHTS^®^.

The investigations relate to an extruder size of d = 35 mm. Larger diameters should be examined to transfer the findings of this work. Additionally, a temperature-dependent contact model for the numerical simulation would be beneficial. Ideally, the temperature in the DEM will be calculated through dissipation between the particles rather than indirectly by the contact model. Trippe’s investigations [24] may be helpful in this regard.

## Figures and Tables

**Table 1:** Overview of applied materials and particle geometry transformation in the numerical simulation environment.

PE-HD Virgin	PE-HD Regrind	PP Virgin	PP Regrind
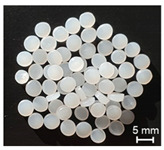	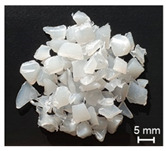	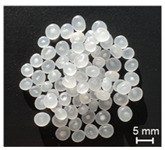	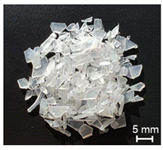
superquadric 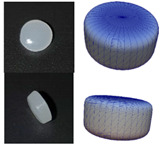	superquadric 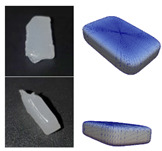	superquadric 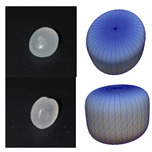	superquadric 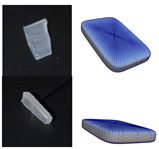
sphere 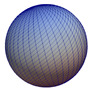	sphere 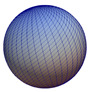	sphere 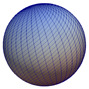	sphere 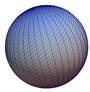

**Table 2:** Material parameters for the numerical simulation.

Simulation Parameter	PE-HD	PP
density in g/cm³	0.945 ^1^	0.91 ^1^
Young’s modulus in MPa	850 ^1^	1850 ^1^
Poisson’s ratio	0.443 ^2^	0.399 ^2^
internal coefficient of restitution	0.87 ^2^	0.81 ^2^
external coefficient of restitution	0.83 ^2^	0.85 ^2^
internal friction	0.498 ^2^	0.432 ^2^
external friction	0.303 ^2^	0.304 ^2^

^1^ from data sheet, ^2^ determined in Thieleke [52].

## Data Availability

Not applicable.

## Appendix B

**Table A1:** Material parameters for the numerical simulation.

	PE-HD Virgin	PE-HD Regrind	PP Virgin	PP Regrind
P12,start	11.04/6182	18.64/4141	7.32/5625	16.68/5099
P3	20.20/47,713	28.13/47,713	20.40/47,713	31.40/47,713
P12,new	7.54/6182	16.96/4141	6.59/5625	19.66/5099
K11	0.62	0.32	0.72	9.16
K12	8.09	20.67	4.19	0.24
K21	10.68	27.28	6.37	0.36
K22	0.82	0.42	1.10	13.92
